# Недостаток белка GAGA у мутантов Trl вызывает массовую клеточную гибель в спермато- и оогенезе дрозофилы

**DOI:** 10.18699/VJ21.033

**Published:** 2021-05

**Authors:** N.V. Dorogova, A.E. Zubkova, Е.V. Fedorova, Е.U. Bolobolova, Е.М. Baricheva

**Affiliations:** Institute of Cytology and Genetics of the Siberian Branch of the Russian Academy of Sciences, Novosibirsk, Russia; Institute of Cytology and Genetics of the Siberian Branch of the Russian Academy of Sciences, Novosibirsk, Russia Novosibirsk State University, Novosibirsk, Russia; Institute of Cytology and Genetics of the Siberian Branch of the Russian Academy of Sciences, Novosibirsk, Russia; Institute of Cytology and Genetics of the Siberian Branch of the Russian Academy of Sciences, Novosibirsk, Russia; Institute of Cytology and Genetics of the Siberian Branch of the Russian Academy of Sciences, Novosibirsk, Russia

**Keywords:** Drosophila, GAGA factor, germ cells, apoptosis, autophagy, spermatogenesis, oogenesis, дрозофила, GAGA-фактор, клетки зародышевого пути, апоптоз, аутофагия, сперматогенез, оогенез

## Abstract

Белок дрозофилы GAGA (GAF) является фактором эпигенетической регуляции транскрипции большой группы генов с широким разнообразием клеточных функций. GAF кодируется геном Trithorax-like (Trl), который экспрессируется в различных органах и тканях на всех стадиях онтогенеза дрозофилы. Мутации этого гена вызывают множественные нарушения развития. В предыдущих работах мы показали, что этот белок необходим для развития половой системы как самцов, так и самок дрозофилы. Снижение экспрессии гена Trl приводило ко множественным нарушениям спермато- и оогенеза. Одно из значительных нарушений было связано с массовой деградацией и потерей клеток зародышевого пути, что позволило предположить, что этот белок вовлечен в регуляцию клеточной гибели. В представленной работе мы провели более детальное цитологическое исследование, чтобы определить, какой тип гибели клеток зародышевого пути характерен для Trl-мутантов, и происходят ли нарушения или изменения этого процесса по сравнению с нормой. Полученные результаты показали, что недостаток белка GAF вызывает массовую гибель клеток зародышевого пути как у самок, так и самцов дрозофилы, но проявляется эта гибель в зависимости от пола по-разному. У самок, мутантных по гену Trl, фенотипически этот процесс не отличается от нормы и в гибнущих яйцевых камерах выявлены признаки апоптоза и аутофагии клеток зародышевого пути. У самцов, мутантных по гену Trl, в отличие от самок, не обнаружены признаки апоптоза. У самцов мутации Trl индуцируют массовую гибель клеток через аутофагию, что не характерно для сперматогенеза дрозофилы и не описано ранее ни в норме, ни у мутаций по другим генам. Таким образом, недостаток GAF у мутантов Trl приводит к усилению апоптотической и аутофагической гибели клеток зародышевого пути. Эктопическая клеточная гибель и атрофия зародышевой линии, вероятно, связаны с нарушением экспрессии генов-мишеней GAGAфактора, среди которых есть гены, регулирующие как апоптоз, так и аутофагию.

## Abstract (en)

Drosophila protein GAGA (GAF) is a factor of epigenetic transcription regulation of a large group of genes with a wide variety of cellular functions. GAF is encoded by the Trithorax-like (Trl) gene, which is important for the formation of various organs and tissues at all stages of ontogenesis. In our previous works, we showed that this protein is necessary for the development of the reproductive system, both in males and females of Drosophila. Decreased expression of the Trl gene led to multiple disorders of spermatogenesis and oogenesis. One of the significant disorders was associated with massive degradation and loss of cells in the germline. In this work, we carried out a more detailed cytological study to determine what type of germ cell death is characteristic of Trl mutants, and whether there are disturbances or changes in this process compared to the norm. The results obtained showed that the lack of GAF protein causes massive germ cell death in both females and males of Drosophila, but this death manifests itself in different ways, depending on the sex. In Trl females, this process does not differ phenotypically from the norm. In the dying egg chambers, signs of apoptosis and autophagy were revealed, as well as morphological features that are characteristic of the wild type. In males, Trl mutations induce mass germ cell death through autophagy, which is not typical of Drosophila spermatogenesis, and has not been previously described, neither in the norm nor in other genes’ mutations. Thus, GAF lack in Trl mutants leads to increased germ cell death through apoptosis and autophagy. Ectopic cell death and germ line atrophy are probably associated with impaired expression of the GAGA factor target genes, among which there are genes that regulate both apoptosis and autophagy.

## Введение

Белок дрозофилы, GAGA-фактор (GAGA-factor, GAF), кодируется геном Trithorax-like (Trl ), который экспрессируется в различных органах и тканях на всех стадиях онтогенеза дрозофилы (Soeller et al., 1993; Baricheva et al., 1997; Karagodin et al., 2013). GAF имеет важную биологическую функцию, связанную как с позитивной, так и негативной регуляцией экспрессии большой группы генов, контролирующих основные этапы развития дрозофилы (Granok et al., 1995; van Steensel et al., 2001, 2003). GAF является эволюционно-консервативным белком, имеющим гомологию с белками многих эукариот, в том числе высших позвоночных (Matharu et al., 2010; Berger, Dubreucq, 2012). Гомологи GAF, как и сам белок, могут связываться с GA-последовательностями в регуляторных районах эволюционно-консервативных генов (Matharu et al., 2010). Результаты полногеномного анализа (профилирование хроматина с использованием Dam) показали, что мишенями GAF у дрозофилы могут быть около 250 генов, участвующих как минимум в 28 сигнальных путях (van Steensel et al., 2003). Анализ профилей связывания GAF, полученных в рамках проекта modENCODE (http://www. modencode.org/) (Roy et al., 2010), позволяет предполагать, что GAF может участвовать в регуляции более 3700 генов дрозофилы (неопубликованные данные).

Анализ Trl-мутантов продемонстрировал, что GAF необходим для эмбриогенеза, развития глаза и крыла дрозофилы (Bhat et al., 1996; Dos-Santos et al., 2008; Omelina et al., 2011; Bayarmagnai et al., 2012).

В предыдущих работах мы показали, что GAF принимает участие в развитии половой системы как самцов, так и самок дрозофилы, поскольку снижение экспрессии гена у мутантов Trl приводит к множественным нарушениям спермато- и оогенеза (Ogienko et al., 2006, 2008; Dorogova et al., 2014; Fedorova et al., 2019). Одно из значительных нарушений как оогенеза, так и сперматогенеза связано с массовой гибелью и потерей клеток зародышевого пути (КЗП) (Dorogova et al., 2014; Fedorova et al., 2019). Это позволило прийти к заключению, что GAF может регулировать активность генов, отвечающих за клеточную гибель. Однако для дальнейшего исследования роли GAF и поиска его генов-мишеней в клеточной гибели необходимо получить более полную информацию о проявлениях этого процесса у Trl-мутантов и соотнести данные с определенным типом регулируемой клеточной смерти согласно существующей в настоящее время классификации. Чтобы решить эту задачу, мы провели детальное цитологическое исследование характера гибели КЗП в яичниках и семенниках у Trl-мутантов дрозофилы.

В норме яичник дрозофилы состоит из 15–20 овариол, которые представляют собой цепочку прогрессивно развивающихся яйцевых камер (ЯК), состоящих из 16 КЗП, которые окружены монослоем соматических фолликулярных клеток. Одна из 16 клеток цисты становится яйцеклеткой, остальные – питающими клетками. По размеру и морфологии ЯК оогенез можно условно разделить на 14 стадий (King, 1957; Spradling, 1993; Ogienko et al., 2007). В норме онтогенетически запрограммированная смерть КЗП происходит на трех специфических стадиях: во вновь сформированных цистах (второй район гермария), во время среднего (стадии 7–9) и позднего (стадии 12, 13) оогенеза. При достаточном питании мух смерть клеток в гермарии и на стадиях 7–9 (называемых контрольными точками гибели клеток в оогенезе, англ. сheckpoints) происходит в ответ на аномалии развития и резко увеличивается под влиянием различных стрессов (McCall, 2004; Jenkins et al., 2013). Гибель питающих клеток в позднем оогенезе происходит как часть нормального развития каждого яйца (Jenkins et al., 2013; Peterson et al., 2015; Bolobolova et al., 2020). 

Для оогенеза дрозофилы характерны два основных типа клеточной гибели: апоптоз и аутофагия (McCall, 2004; Barth et al., 2011; Jenkins et al., 2013; Bolobolova et al., 2020). Апоптоз является универсальным консервативным механизмом, при котором запускается программа саморазрушения клеток с участием протеолитических ферментов, относящихся к семейству каспаз (Kumar, 2007). В гибнущих ЯК проявляются характерные признаки апоптоза: конденсация хроматина, разрывы ДНК, фрагментация ядерного материала и цитоплазмы (Kihlmark et al., 2001; Greenwood, Gautier, 2005; Sarkissian et al., 2014). У дрозофилы инициация апоптоза связана с активностью эффекторной каспазы Dcp-1, и окраска антителами к этому белку является основным маркером каспаза-зависимого апоптоза (Sarkissian et al., 2014). Клеточная гибель через аутофагию сопровождается избыточным образованием аутофаго- и лизосом, что приводит к перевариванию всех клеточных органелл и закислению цитоплазмы. Вследствие этой особенности для выявления аутофагии используют лизотрекер (LysoTracker), ацидофильный краситель, который маркирует лизо- и аутофагосомы (DeVorkin, Gorski, 2014).

В семенниках КЗП расположены вдоль органа в соответствии со стадиями сперматогенеза. На апикальном конце – стволовые клетки, которые делятся с образованием гониальных клеток. Гониальные клетки вступают в сперматогенез, который включает митотическое и мейотическое деления, в результате чего образуются 64 синцитиальные сперматиды, которые затем дифференцируются в сперматозоиды (Fuller, 1993; Fabian, Brill, 2012). Клеточная гибель в сперматогенезе дрозофилы происходит крайне редко, и примеры ее исследования – единичны. Показано, что в процессе сперматогенеза дефектные сперматоциты элиминируются с помощью лизосомного механизма (без формирования аутофагосом) (Yacobi-Sharon et al., 2013), а также программируемого некроза, опосредованного белком p53 (Napoletano et al., 2017).

В данной работе мы показали, что снижение экспрессии GAF в яичниках у самок Trl-мутантов приводит к усилению гибели КЗП и деградации ЯК на 7–9-й стадиях оогенеза. При этом процесс клеточной гибели протекает так же, как в норме, и имеет признаки как аутофагии, так и апоптоза. В семенниках недостаток GAF индуцирует массовую гибель через аутофагию, что не характерно для сперматогенеза и не описано ранее ни в норме, ни у мутаций по другим генам.

## Материал и методы

В экспериментах использовали следующие мутации D. melanogaster: мутация TrlR85 – нуль-аллель гена, любезно предоставлена Ф. Каршем (Женевский университет, Швейцария) (Farkas et al., 1994); гипоморфные мутации Trl362 и Trl(ex)15, нарушающие 5′-область гена, получены в ИЦиГ СО РАН (Ogienko et al., 2007; Dorogova et al., 2014); Oregon R – дикий тип, из фонда лаборатории ИЦиГ СО РАН, использована в качестве контроля. Все скрещивания проводили на стандартной среде при температуре 25 °С.

Выделение, фиксацию и окраску гонад для электронной и флуоресцентной микроскопии производили согласно описанной ранее методике (Dorogova et al., 2014). В работе использованы первичные антитела: rabbit anti-Vasa (разведение 1:300; SC30210, Santa Cruz Biotechnology), rabbit anti-Dcp-1 (разведение 1:100; Asp216, Cell Signaling Technology). Вторичные антитела – anti-rabbit, конъюгированные с AlexaFluor-488 (1:500; A-11001, Thermo Fisher Scientific) и AlexaFluor-568 (1:500; A-11369, Thermo Fisher Scientific). Анализ с помощью LysoTracker выполняли, как описано в предыдущей работе (Dorogova et al., 2014) (LysoTracker Red DND-99, Thermo Fisher Scientific). После окраски яичники и семенники помещали в реагент против выцветания ProLong Gold с DAPI (Thermo Fisher Scientific). Изображения получены с помощью микроскопа AxioImager Z1 с приставкой ApoTome (Zeiss), программным обеспечением AxioCam MR и AxioVision (Zeiss).

## Результаты

В предыдущих работах мы представили данные, позволяющие сделать вывод, что снижение экспрессии гена Trl у мутантов приводит к значительному усилению клеточной гибели в спермато- и среднем оогенезе дрозофилы (Dorogova et al., 2014; Fedorova et al., 2019). У самцов и самок дрозофилы, несущих мутантные аллели Trl362 и Trl(ex)15 в сочетании с нуль-аллелем TrlR85 (TrlR85/Trl362 и TrlR85/Trl(ex)15), наблюдалась массовая гибель КЗП (Dorogova et al., 2014; Fedorova et al., 2019). В этой работе, проведя более детальное цитологическое исследование данных аллельных комбинаций, мы определили, какой тип гибели КЗП характерен для Trl-мутантов и как нарушается или меняется этот процесс по сравнению с нормой.

**Мутации Trl приводят к усилению клеточной гибели в оогенезе, но не влияют на цитологические проявления этого процесса**

Для выявления аутофагии мы использовали лизотрекер (LysoTracker), ацидофильный краситель, который маркирует лизо- и аутофагосомы. После окрашивания сигнал лизотрекера был обнаружен во всех деградирующих ЯК, но его появление совпадало с началом ядерной конденсации как в яичниках мух дикого типа, так и у Trl-мутантов (рис. 1, а, б). Таким образом, появление аутофаго- и лизосом сопутствовало уже начавшемуся апоптозу, но не предшествовало ему. Это означает, что клеточная гибель в среднем оогенезе у мутантов Trl не связана с процессами, которые приводят к избыточной аутофагии, несовместимой с жизнеспособностью клеток.

**Fig. 1.**
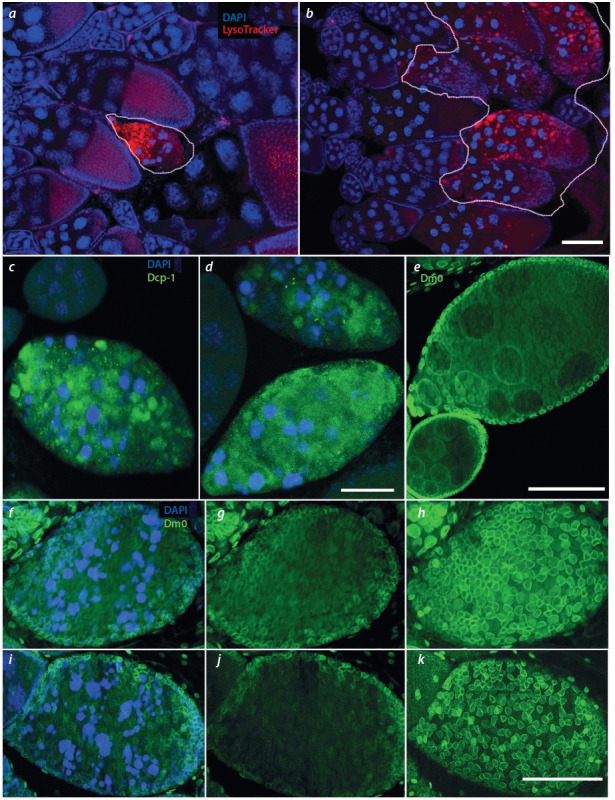
Features of cell death manifestation in the oogenesis of females mutant for Trl (TrlR85/Trl362). a, b – Identification of autophagosomes and lysosome in dying egg chambers using LysoTracker red. a – In Oregon R females ovaries, only few egg chambers undergo cell death (outlined), the rest develop normally and enter vitellogenesis. b – In Trl females, most egg chambers die at stages 7–9 of oogenesis (outlined), but the LysoTracker staining pattern in mutants does not differ from that in Oregon R. c, d – Staining with antibodies to the Dcp-1 effector caspase. Caspase Dcp-1 is detected in the same way in dying egg chambers of (c) Oregon R and (d) Trl. e–k – Staining with antibodies to the Lamin Dm0 protein, allowing the assessment of the nuclear envelope integrity during cell death. e – The nuclear envelope is clearly visible in all cells of the egg chambers that do not undergo degradation. f–h – In the degrading egg chambers of Oregon R females, the nurse cell nuclear membrane is destroyed (g), but it is preserved and visualized in follicular cells (h). i–k – In Trl females ovaries, the pattern of staining with antibodies to caspase Dcp-1 is the same as in Oregon R females. LysoTracker is red; caspase Dcp-1, green; and Lamin Dm0, green. Scale bars: a, b – 15 μm; c–e – 30 μm; f–k – 40 μm.

Для дополнительной верификации апоптоза и выявления активности каспаз мы использовали антитела к эффекторной каспазе Dcp-1. Иммунофлуоресцентное окрашивание антителами к Dcp-1 показало, что этот белок выявляется в гибнущих яйцевых камерах Trl-мутантов и его паттерн не отличается от такового у самок дикого типа (см. рис. 1, в, г). 

Поскольку при апоптозе разрушается и фрагментируется ядерная оболочка, с помощью антител к белку ядерной ламины дрозофилы, Lamin Dm0, мы проверили целостность ядерной оболочки в клетках гибнущих ЯК. Ядерная оболочка четко выявлялась антителами к белку ламины во всех клетках ЯК, не подверженных деградации (см. рис. 1, д). Однако с появлением признаков конденсации хроматина ядерная оболочка не визуализировалась в КЗП как у мутантов, так и в контроле. Эта структура оставалась видимой только в фолликулярных клетках, которые деградируют позже, после того как осуществят фагоцитоз погибших питающих клеток (см. рис. 1, е–л).

Мы также провели анализ деградирующих ЯК методами электронной микроскопии, который выявил специфические изменения в ядре и цитоплазме, характерные для нормального проявления клеточной гибели в среднем оогенезе, описанного ранее в других работах (Giorgi, Deri, 1976) (данные не представлены). То есть у мутантов этот процесс морфологически не отличался от контроля (Oregon R). 

Таким образом, эффект мутации Trl связан с усилением клеточной гибели в среднем оогенезе, что соответствует ранее полученным результатам (Dorogova et al., 2014; Fedorova et al., 2019). Этот процесс становится более массовым у мутантов TrlR85/Trl362 и TrlR85/Trl(ex)15, но основные критерии и морфологические характеристики соответствуют норме.

**Мутации Trl индуцируют массовую аутофагию в сперматогенезе**

Используя такой же методологический подход, мы проанализировали, как происходит гибель КЗП у мутантов Trl в сперматогенезе. Предварительное иммуноокрашивание антителами к белку Vasa, специфичному для КЗП, показало, что у мутантов выявляются преимущественно ранние стадии сперматогенеза. Большинство цист на более поздних стадиях сперматогенеза элиминируются в процессе гибели (рис. 2, а, б). Однако особенностью гибнущих КЗП в сперматогенезе было сохранение целостности ядерной оболочки. Ядерная оболочка визуализируется антителами к Lamin Dm0 даже в сперматоцитах, которые уже не окрашиваются антителами к Vasa (см. рис. 2, а, б).

**Fig. 2.**
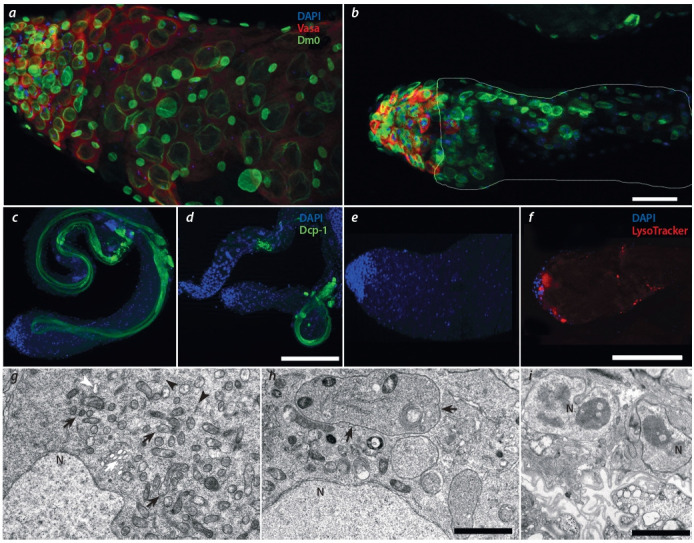
Features of cell death in the spermatogenesis of Trl mutant males (в TrlR85/Trl362) a, b – Immunostaining with antibodies to the Vasa and Lamin Dm0 proteins of the testicle regions where germ tract cells are located at the early stages of spermatogenesis. a – In wild-type males, the entire basal region of the testis is filled with spermatocytes at early stages of spermatogenesis, which are stained with antibodies to the Vasa protein. b – In mutants, germ cells are detected only in the apical region, and all of them are at the very beginning of spermatogenesis. The nuclear membrane is visualized with antibodies to Lamin Dm0 even in spermatocytes that are no longer stained with antibodies to Vasa. c, d – The staining pattern with antibodies to the Dcp-1 protein corresponds to the late stages of spermatogenesis, both in (c) Oregon R males and (d) mutants. In mutants, Dcp-1 is not detected in testis regions where mass-scale cell death occurs (d). e, f – Detection of lysosome and autophagosome activity with LysoTracker. The LysoTracker signal is much more intense in Trl (f) than in the wild type (e). g–i – Ultrastructure of spermatocytes at the interphase (before meiosis) in the wild type (g) and in the mutant (h, i). g – In normal spermatogenesis, numerous mitochondria (black arrow), membranes of the endoplasmic reticulum (black arrowhead), multivesicular bodies (white arrow), and occasional lysosomes (white arrowhead) are observed in the cells at this stage. h – Mutant cytoplasm is filled with autophagosomes (arrow). i – Spermatocytes at the lysis stage retain the internal structure of the nucleus and the nuclear membrane (arrow). Vasa and LysoTracker are red; caspase Dcp-1 and Lamin Dm0, green; N – nucleus. Scale bars: a, b – 20 μm; c, d – 30 μm; e, f – 20 μm; g, h – 1 μm; i – 3 μm.

У мутантных самцов, в отличие от самок, в генеративной ткани не обнаружены признаки апоптоза. Окрашивание антителами к белку Dcp-1 показало активность эффекторной каспазы только на самых поздних стадиях сперматогенеза, во время которых в процессе, называемом индивидуализацией, образуются зрелые сперматозоиды. Такой же паттерн окрашивания наблюдался и в контроле (см. рис. 2, в, г). В районах семенника, где у мутантных самцов происходит гибель КЗП, каспаза Dcp-1 не выявлялась. Окрашивание DAPI также подтверждает, что хроматин в этих клетках не подвергается конденсации и фрагментации, характерных для апоптоза.

Окраска лизотрекером показала, что ацидофильные компартменты, соответствующие аутофаго- и лизосомам, в изобилии присутствуют во внутренней области семенников. Эти структуры располагаются преимущественно в зоне семенника, где находятся КЗП на ранних стадиях развития – сперматоциты первого порядка (см. рис. 2, д, е).

С помощью электронно-микроскопического анализа в цитоплазме гибнущих сперматоцитов выявлялись множественные аутофаго- и лизосомы, при этом ядерная оболочка не разрушалась и морфология ядер не отличалась от таковой в негибнущих КЗП (см. рис. 2, ж–и).

В результате сравнения проявлений клеточной гибели в сперматогенезе у самцов TrlR85/Trl362 и TrlR85/Trl(ex)15 не обнаружено фенотипических отличий. Обе мутации вызывают массовую аутофагию и последующий лизис КЗП. Однако процесс гибели этих клеток не сопровождается апоптозом. В норме, у линии Oregon R, не выявлены ни признаки апоптоза, ни аутофагии. 

## Обсуждение

Полученные результаты показали, что недостаток белка GAF вызывает массовую гибель КЗП как у самок, так и самцов дрозофилы, но проявляется эта гибель в зависимости от пола по-разному

В оогенезе у самок, мутантных по гену Trl, большинство ЯК имеют низкую жизнеспособность и не могут пройти через контрольную точку среднего оогенеза (mid oogenesis check point). Известно, что эта стадиоспецифичная контрольная точка активируется в ответ на неблагоприятные стимулы, физиологические нарушения или патологии развития. Яйцевые камеры, которые не пропускаются на следующий этап оогенеза (вителлогенез), подвергаются генетически регулируемой клеточной гибели (Pritchett et al., 2009; Jenkins et al., 2013; Peterson et al., 2015). Отличительной чертой мутантного фенотипа был только высокий показатель гибнущих ЯК, при этом деградируют они так же, как в диком типе. В гибнущих ЯК обнаружены признаки апоптоза и аутофагии, а также морфологические изменения, характерные для нормы. 

Согласно данным литературы, подобный фенотип может возникать в ответ на недостаток питательных веществ или снижение активности компонентов инсулин/ TOR-сигнального пути (Drummond-Barbosa, Spradling, 2001; Barth et al., 2011; Pritchett, McCall, 2012). Инсулин/ TOR-сигнальный путь – консервативный механизм, ответственный за рост клеток и тканей. Он действует как сенсор доступности питательных веществ, способствуя метаболизму, росту и пролиферации клеток. В оогенезе Drosophila этот механизм является критически важным для развития КЗП и созревания ооцитов. При его нарушениях ЯК не могут вступать в энергоемкий вителлогенез и деградируют (LaFever et al., 2010; Laws, Drummond-Barbosa, 2017; Jeong et al., 2019). Массовая деградация ЯК в среднем оогенезе также наблюдалась при подавлении экспрессии генов, кодирующих белки, принадлежащие семейству ингибиторов апоптоза – Bruce и Diap, которые негативно регулируют активность каспаз. В таком случае происходило усиление клеточной гибели на фоне нарушения ее регуляции, при этом морфологические критерии этого процесса не менялись и не отличались от нормы (Rodriguez et al., 2002; Xu et al., 2005; Hou et al., 2008). У Trl-мутантов отмечен похожий фенотип в оогенезе, что позволяет предположить нарушение регуляторного механизма клеточной гибели. 

В сперматогенезе у Trl-мутантов массовая гибель КЗП реализуется через механизм избыточной аутофагии. При нормальном развитии зародышевой линии дефектные сперматоциты периодически элиминируются накануне мейоза с помощью лизосом и катаболических ферментов без участия аутофагосом (Yacobi-Sharon et al., 2013). Этот механизм является одним из вариантов генетически регулируемой клеточной гибели и включен в последний каталог Комитета по номенклатуре клеточной смерти (Nomenclature Committee on Cell Death) (Galluzzi et al., 2018). У Trl-мутантов лизосомы также принимают участие в деградации сперматоцитов, однако появляются на заключительном этапе гибели, после того как цитоплазма клеток заполнится аутофагосомами. 

Важно отметить, что основная функция аутофагии не убивать, а защищать клетки. С ее помощью происходит удаление из клеток поврежденных и состарившихся органелл, цитоплазматических фрагментов, неправильных или нефункциональных белков (Denton et al., 2013; Fitzwalter, Thorburn, 2015; Swart et al., 2016). Базальный (репаративный) уровень аутофагии необходим для поддержания нормальных физиологических условий функционирования клеток (Glick et al., 2010). При определенных условиях, связанных со спецификой развития или стрессовыми воздействиями, аутофагия становится массовой и вместо цитопротекторной функции индуцирует клеточную гибель (Fitzwalter, Thorburn, 2015; Swart et al., 2016). Поэтому аутофагия включена в каталог клеточной смерти как одна из основных форм (Galluzzi et al., 2012, 2018).

У дрозофилы клеточная гибель через аутофагию обнаружена при деградации слюнных желез и средней кишки на стадии метаморфоза «личинка–куколка». Атрофия этих органов является онтогенетически программируемой и регулируется одним и тем же стероидным гормоном – экдизоном (Berry, Baehrecke, 2007; Denton et al., 2012). Однако избыточная аутофагия в сперматогенезе у Trl-мутантов, вероятно, не связана с экдизоном, поскольку в сперматоцитах не были выявлены активные рецепторы к этому гормону (Schwedes et al., 2011). Аутофагии и лизису подвергались сперматоциты на стадии роста перед мейотическим делением. Для этой стадии характерен высокий уровень транскрипции генов и синтетической активности. В норме объем сперматоцитов возрастает в 25 раз, что требует значительного потребления энергии и ресурсов (Fuller, 1993). Можно предположить, что мутации Trl негативно влияют на клеточный метаболизм, не позволяя достичь необходимого уровня синтеза макромолекул и роста клеток. В результате активируется сигнальный путь, регулируемый TOR-киназой, который индуцирует клеточную гибель через аутофагию. Однако также можно предположить, что недостаток белка GAF приводит к нарушению экспрессии генов, кодирующих компоненты TOR-зависимого сигнального пути или/и факторы, регулирующие аутофагию, что также может вызвать эктопическую гибель.

Известно, что GAGA-фактор участвует в регуляции транскрипции большой группы генов с различными клеточными функциями (van Steensel et al., 2003; Omelina et al., 2011). В базе данных Flybase аннотированы гены, участвующие в широком спектре процессов, связанных с клеточной гибелью (Gene Ontology terms: apoptotic process, autophagic cell death, salivary gland cell autophagic death). Всего в этой группе представлено около 400 генов, приблизительно 180 из них, согласно профилям связывания GAF из проекта mоdeNCODE, содержат в промоторных районах сайты связывания GAF и являются, таким образом, потенциальными мишенями этого белка. Наибольший интерес представляют присутствующие в этом списке консервативные гены аутофагии – Atg2, Atg4, Atg5, Atg7, Atg8, Atg9, Atg16, Atg17, Atg18. Большинство этих генов кодируют белки, непосредственно участвующие в формировании и созревании аутофагосом, а Atg17 – белок, контролирующий инициацию аутофагии (Noda, Inagaki, 2015). Также потенциальным геном-мишенью GAF является Tor, кодирующий киназу, вовлеченную в регуляцию аутофагии. Инактивация TOR-киназы в ответ на недостаток питательных веществ или ростовых факторов стимулирует аутофагию (Levine, Klionsky, 2004; Das et al., 2012). Если предположить, что GAF регулирует Tor и группу генов Atg в сперматогенезе, то его недостаток может вызвать нарушение экспрессии этих генов и привести к массовой и неуправляемой аутофагии. При этом по отношению к Atg-генам GAF должен выполнять функцию негативной регуляции, а по отношению к Tor – позитивной.

В оогенезе у мутантов Trl массовая клеточная гибель происходит не только за счет аутофагии, но и апоптоза, поэтому очевидно, что в этом случае дополнительно может нарушаться активность генов, регулирующих апоптоз. Одним из вероятных кандидатов является ген diap (deathassociated inhibitor of apoptosis), который, помимо того что является потенциальной мишенью GAF, в мутантной форме индуцирует такой же фенотип в оогенезе, как и Trl (Rodriguez et al., 2002; Xu et al., 2005).

## Conclusion

Фенотип массовой гибели КЗП у Trl-мутантов имеет определенную специфику проявления в спермато- и оогенезе. В яичниках аутофагия сопутствует апоптозу, что соответствует каноническому сценарию клеточной гибели в среднем оогенезе дрозофилы. В семенниках наблюдается не характерная для этого типа ткани гибель через аутофагию, которая становится экспансивной и является основной причиной атрофии зародышевой линии. Массовая потеря КЗП связана с недостатком GAGA-фактора, что, вероятно, приводит к нарушению экспрессии геновмишеней этого белка, отвечающих за клеточную гибель. К его потенциальным мишеням относятся как гены аутофагии, так и апоптоза, и можно предположить, что обе эти группы зависят от активности GAF в оогенезе. Тогда как в сперматогенезе этот белок взаимодействует только с генами аутофагии. Чтобы установить, какие гены под контролем GAF вовлечены в разные механизмы клеточной гибели, необходимо привлечь транскриптомные технологии и проанализировать изменения профилей экспрессии генов в спермато- и оогенезе на фоне недостатка GAF у Trl-мутантов. 

## Conflict of interest

The authors declare no conflict of interest.

## References

Baricheva E.M., Katokhin A.V., Perelygina L.M. Expression of Drosophila melanogaster gene encoding transcription factor GAGA is
tissue-specific and temperature-dependent. FEBS Lett. 1997;414(2):
285-288. DOI 10.1016/s0014-5793(97)01010-7.

Barth J.M., Szabad J., Hafen E., Köhler K. Autophagy in Drosophila
ovaries is induced by starvation and is required for oogenesis. Cell
Death Differ. 2011;18(6):915-924. DOI 10.1038/cdd.2010.157.

Bayarmagnai B., Nicolay B.N., Islam A.B., Lopez-Bigas N., Frolov M.V. Drosophila GAGA factor is required for full activation of
the dE2f1-Yki/Sd transcriptional program. Cell Cycle. 2012;11(22):
4191-4202. DOI 10.4161/cc.22486.

Berger N., Dubreucq B. Evolution goes GAGA: GAGA binding proteins across kingdoms. Biochim. Biophys. Acta. 2012;1819(8):863-
868. DOI 10.1016/j.bbagrm.2012.02.022.

Berry D.L, Baehrecke E.H. Growth arrest and autophagy are required
for salivary gland cell degradation in Drosophila. Cell. 2007;131:
1137-1148. DOI 10.1016/j.cell.2007.10.048.

Bhat K.M., Farkas G., Karch F., Gyurkovics H., Gausz J., Schedl P. The
GAGA factor is required in the early Drosophila embryo not only
for transcriptional regulation but also for nuclear division. Development. 1996;122;(4):1113-1124.

Bolobolova E.U., Dorogova N.V., Fedorova S.A. Major scenarios of
genetically regulated cell death during oogenesis in Drosophila melanogaster. Russ. J. Genet. 2020;56:655-665. DOI 10.1134/S10227
95420060034.

Das G., Shravage B.V., Baehrecke E.H. Regulation and function of
autophagy during cell survival and cell death. Cold Spring Harb.
Perspect. Biol. 2012;4(6):a008813. DOI 10.1101/cshperspect.a00
8813.

Denton D., Aung-Htut M.T., Lorensuhewa N., Nicolson S., Zhu W.,
Mills K., Cakouros D., Bergmann A., Kumar S. UTX coordinates
steroid hormone-mediated autophagy and cell death. Nat. Commun.
2013;4:2916. DOI 10.1038/ncomms3916.

Denton D., Chang T.-K., Nicolson S., Shravage B., Simin R., Baehrecke E.H., Kumar S. Relationship between growth arrest and autophagy in midgut programmed cell death in Drosophila. Cell Death
Differ. 2012;19:1299-1307. DOI 10.1038/cdd.2012.43.

DeVorkin L., Gorski S.M. LysoTracker staining to aid in monitoring
autophagy in Drosophila. Cold Spring Harb. Protoc. 2014;9:951-
958. DOI 10.1101/pdb.prot080325.

Dorogova N.V., Fedorova E.V., Bolobolova E.U., Ogienko A.A., Baricheva E.M. GAGA protein is essential for male germ cell development in Drosophila. Genesis. 2014;52(8):738-751. DOI 10.1002/
dvg.22789.

Dorogova N.V., Khrushcheva A.S., Fedorova E.V., Ogienko A.A., Baricheva E.M. Role of GAGA factor in Drosophila primordial germ
cell migration and gonad development. Ontogenez. 2016;47(1):40-48.

Dos-Santos N., Rubin T., Chalvet F., Gandille P., Cremazy F., Leroy J.,
Boissonneau E., Theodore L. Drosophila retinal pigment cell death
is regulated in a position-dependent manner by a cell memory gene.
Int. J. Dev. Biol. 2008;52(1):21-31. DOI 10.1387/ijdb.072406nd.

Drummond-Barbosa D., Spradling A.C. Stem cells and their progeny
respond to nutritional changes during Drosophila oogenesis. Dev.
Biol. 2001;231:265-278. DOI 10.1006/dbio.2000.0135.

Fabian L., Brill J.A. Drosophila spermiogenesis: Big things come from
little packages. Spermatogenesis. 2012;2(3):197-212. DOI 10.4161/
spmg.21798.

Farkas G., Gausz J., Galloni M., Reuter G., Gyurkovics H., Karch F.
The Trithorax-like gene encodes the Drosophila GAGA factor.
Nature. 1994;371:806-808. DOI 10.1038/371806a0.

Fedorova E.V., Dorogova N.V., Bolobolova E.U., Fedorova S.A.,
Karagodin D.A., Ogienko A.A., Khruscheva A.S., Baricheva E.M.
GAGA protein is required for multiple aspects of Drosophila
oogenesis and female fertility. Genesis. 2019;57(2):e23269. DOI
10.1002/dvg.23269.

Fitzwalter B., Thorburn A. Recent insights into cell death and autophagy. FEBS J. 2015;282(22):4279-4288. DOI 10.1111/febs.13515.

Fuller M.T. Spermatogenesis In: The development of Drosophila melanogaster. NY Cold Spring Harbor. Press, 1993;71-147.

Galluzzi L., Vitale I., Aaronson S.A., Abrams J.M., Adam D., Agostinis P., Alnemri E.S., Altucci L., Amelio I., Andrews D.W., …,
Zakeri Z., Zhivotovsky B., Zitvogel L., Melino G., Kroemer G.
Molecular mechanisms of cell death: recommendations of the Nomenclature Committee on Cell Death 2018. Cell Death Differ. 2018;
25(3):486-541. DOI 10.1038/s41418-017-0012-4.

Galluzzi L., Vitale I., Abrams J.M., Alnemri E.S., Baehrecke E.H.,
Blagosklonny M.V., Dawson T.M., Dawson V.L., El-Deiry W.S.,
Fulda S., Gottlieb E., Green D.R., Hengartner M.O., Kepp O.,
Knight R.A., Kumar S., Lipton S.A., Lu X., Madeo F., Malorni W.,
Mehlen P., Nuñez G., Peter M.E., Piacentini M., Rubinsztein D.C.,
Shi Y., Simon H.-U., Vandenabeele P., White E., Yuan J., Zhivotovsky B., Melino G., Kroemer G. Molecular definitions of cell death
subroutines: recommendations of the Nomenclature Committee on
Cell Death 2012. Cell Death Differ. 2012;19:107-120. DOI 10.1038/
cdd.2011.96.

Giorgi F., Deri P. Cell death in ovarian chambers of Drosophila melanogaster. J. Embryol. Exp. Morphol. 1976;35:521-533.
Glick D., Barth S., Macleod K.F. Autophagy: cellular and molecular mechanisms. J. Pathol. 2010;221(1):3-12. DOI 10.1002/path.
2697.

Granok H., Leibovitch B.A., Shaffer C.D., Elgin S.C.R. Gaga over
GAGA factor. Curr. Biol. 1995;5:238-241. DOI 10.1016/s0960-
9822(95)00048-0.

Greenwood J., Gautier J. From oogenesis through gastrulation: developmental regulation of apoptosis. Semin. Cell Dev. Biol. 2005;16:215-
224. DOI 10.1016/j.semcdb.2004.12.002.

Hay B.A., Guo M. Caspase-dependent cell death in Drosophila. Annu.
Rev. Cell Dev. Biol. 2006;22:623-650. DOI 10.1146/annurev.cellbio.
21.012804.093845.

Hou Y.C., Chittaranjan S., Barbosa S.G., McСall K., Gorski S.M. Effector caspase Dcp-1 and IAP protein Bruce regulate starvation-induced
autophagy during Drosophila melanogaster oogenesis. J. Cell Biol.
2008;182:1127-1139. DOI 10.1083/jcb.200712091.

Jenkins V.K., Timmons A.K., McСall K. Diversity of cell death pathways: insight from the fly ovary. Trends Cell Biol. 2013;23(11):567-
574. DOI 10.1016/j.tcb.2013.07.005.

Jeong E.B., Jeong S.S., Cho E., Kim E.Y. Makorin 1 is required for
Drosophila oogenesis by regulating insulin/Tor signaling. PLoS
One. 2019;14(4):e0215688. DOI 10.1371/journal.pone.0215688.

Karagodin D.A., Omelina E.S., Fedorova E.V., Baricheva E.M. Identification of functionally significant elements in the second intron of
the Drosophila melanogaster Trithorax-like gene. Gene. 2013;520:
178-184. DOI 10.1016/j.gene.2013.02.012.

Kihlmark M., Imreh G., Hallberg E. Sequential degradation of proteins from the nuclear envelope during apoptosis. J. Cell Sci. 2001;
114(Pt 20):3643-3653.

King R.C. Ovarian development in Drosophila melanogaster. New
York: Academic Press Inc., 1970.

Kumar S. Caspase function in programmed cell death. Cell Death Differ. 2007;14(1):32-43. DOI 10.1038/sj.cdd.4402060.

LaFever L., Feoktistov A., Hsu H.J., Drummond-Barbosa D. Specific
roles of Target of rapamycin in the control of stem cells and their
progeny in the Drosophila ovary. Development. 2010;137:2117-
2126. DOI 10.1242/dev.050351.

Laws K.M., Drummond-Barbosa D. Control of germline stem cell lineages by diet and physiology. Results Probl. Cell Differ. 2017;59:
67-99. DOI 10.1007/978-3-319-44820-6_3.

Levine B., Klionsky D.J. Development by self-digestion: molecular
mechanisms and biological functions of autophagy. Dev. Cell. 2004;
6:463‑477. DOI 10.1016/s1534-5807(04)00099-1.

Matharu N.K., Hussain T., Sankaranarayanan R., Mishra R.K. Vertebrate homologue of Drosophila GAGA factor. J. Mol. Biol. 2010;
400:434-447. DOI 10.1016/j.jmb.2010.05.010.

McCall K. Eggs over easy: cell death in the Drosophila ovary. Dev.
Biol. 2004;274(1):3-14. DOI 10.1016/j.ydbio.2004.07.017.

Napoletano F., Gibert B., Yacobi-Sharon K., Vincent S., Favrot C.,
Mehlen P., Girard V., Teil M., Chatelain G., Walter L., Arama E.,
Mollereau B. p53-dependent programmed necrosis controls germ
cell homeostasis during spermatogenesis. PLoS Genet. 2017;13(9):
e1007024. DOI 10.1371/journal.pgen.1007024.

Noda N.N., Inagaki F. Mechanisms of autophagy Annu. Rev. Biophys. 2015;44:101-122. DOI 10.1146/annurev-biophys-060414-
034248.

Ogienko A.A., Fedorova S.A., Baricheva E.M. Basic aspects of ovarian development in Drosophila melanogaster. Russ. J. Genet. 2007;
43(10):1120-1134.

Ogienko A.A., Karagodin D.A., Fedorova S.A., Fedorova E.V., Lashina V.V., Baricheva E.M. Effect of hypomorphic mutation in Trithorax-like gene on Drosophila melanogaster oogenesis. Ontogenez.
2006;37(3):211-220.

Ogienko A.A., Karagodin D.A., Pavlova N.V., Fedorova S.A., Voloshina M.A., Baricheva E.M. Molecular and genetic description of
a new hypomorphic mutation of Trithorax-like gene and analysis of
its effect on Drosophila melanogaster oogenesis. Ontogenez. 2008;
39(2):134-142.

Omelina E.S., Baricheva E.M., Oshchepkov D.Y., Merkulova T.I. Analysis and recognition of the GAGA transcription factor binding sites
in Drosophila genes. Comput. Biol. Chem. 2011;35(6):363-370.
DOI 10.1016/j.compbiolchem. 2011.10.008.

Omichinski J.G., Pedone P.V., Felsenfeld G., Gronenborn A.M., Clore G.M. The solution structure of a specific GAGA factor-DNA
complex reveals a modular binding mode. Nat. Struct. Biol. 1997;4:
122-132.

Peterson J.S., Timmons A.K., Mondragon A.A., McСall K. The end of
the beginning: cell death in the germline. Curr. Top. Dev. Biol. 2015;
114:93-119. DOI 10.1016/bs.ctdb.2015.07.025.

Pritchett T.L., McСall K. Role of the insulin/Tor signaling network
in starvation-induced programmed cell death in Drosophila oogenesis. Cell Death Differ. 2012;19:1069-1079. DOI 10.1038/cdd.
2011.200.

Pritchett T.L., Tanner E.A., McСall K. Cracking open cell death in
the Drosophila ovary. Apoptosis. 2009;14:969-979. DOI 10.1007/
s10495-009-0369-z.

Rodriguez A., Chen P., Oliver H., Abrams J.M. Unrestrained caspasedependent cell death caused by loss of Diap1 function requires the
Drosophila Apaf-1 homolog, Dark. EMBO J. 2002;21:2189-2197.
DOI 10.1093/emboj/21.9.2189.

Roy S., Ernst J., Kharchenko P.V., Kheradpour P., Negre N., Eaton M.L.,
Landolin J.M., Bristow C.A., Ma L.J., Lin M.F., Kellis M. Identification of functional elements and regulatory circuits by Drosophila
modENCODE. Science. 2010;330(6012):1787-1797. DOI 10.1126/
science.1198374.

Sarkissian T., Timmons A., Arya R., Abdelwahid E., White K. Detecting apoptosis in Drosophila tissues and cells. Methods. 2014;68:
89-96. DOI 10.1016/j.ymeth.2014.02.033.

Schwedes C., Tulsiani S., Carney G.E. Ecdysone receptor expression
and activity in adult Drosophila melanogaster. J. Insect Physiol.
2011;57(7):899-907. DOI 10.1016/j.jinsphys.2011.03.027.

Soeller W.C., Oh C.E., Kornberg T.B. Isolation of cDNAs encoding
the Drosophila GAGA transcription factor. Mol. Cell. Biol. 1993;13:
7961-7970. DOI 10.1128/mcb.13.12.7961.

Spradling A.C. Developmental genetics of oogenesis. The development
of Drosophila melanogaster. NY Cold Spring Harb. Lab. Press,
1993;1-70. 

Swart Ch., Du Toit A., Loos B. Autophagy and the invisible line between life and death. Eur. J. Cell Biol. 2016;95(12):598-610. DOI
10.1016/j.ejcb.2016.10.005.

van Steensel B., Delrow J., Bussemaker H.J. Genomewide analysis of
Drosophila GAGA factor target genes reveals context-dependent
DNA binding. Proc. Natl. Acad. Sci. USA. 2003;100(5):2580-2580.
DOI 10.1073/pnas.0438000100.

van Steensel B., Delrow J., Henikoff S. Supplementary information:
Chromatin profiling using targeted DNA adenine methyltransferase.
Nat. Genet. 2001;27(3):304-308. DOI 10.1038/85871.

Wilkins R.C., Lis J.T. Dynamics of potentiation and activation: GAGA
factor and its role in heat shock gene regulation. Nucleic Acids Res.
1997;25(20):3963-3968. DOI 10.1093/nar/25.20.3963.

Xu D., Li Y., Arcaro M., Lackey M., Bergmann A. The CARD-carrying caspase Dronc is essential for most, but not all, developmental
cell death in Drosophila. Development. 2005;132:2125-2134. DOI
10.1242/dev.01790.

Yacobi-Sharon K., Namdar Y., Arama E. Alternative germ cell death
pathway in Drosophila involves HtrA2/Omi, lysosomes, and a caspase-9 counterpart. Dev Cell. 2013;25:29-42. DOI 10.1016/j.devcel.
2013.02.002.

